# Face specific neural anticipatory activity in infants 4 and 9 months old

**DOI:** 10.1038/s41598-022-17273-1

**Published:** 2022-07-28

**Authors:** Giovanni Mento, Gian Marco Duma, Eloisa Valenza, Teresa Farroni

**Affiliations:** 1 Department of General Psychology, University of Padova, Italy Via Venezia, 8, 35131 Padua, PD Italy; 2 Padua Neuroscience Center (PNC), University of Padova, Padua, Italy; 3 Department of Developmental Psychology and Socialization, University of Padova, Padua, Italy; 4 Institut de Neurosciences des Systèmes, University of Aix-Marseille, Marseille, France

**Keywords:** Neuroscience, Cognitive neuroscience, Development of the nervous system, Sensory processing, Social neuroscience, Psychology

## Abstract

The possibility of predicting the specific features of forthcoming environmental events is fundamental for our survival since it allows us to proactively regulate our behaviour, enhancing our chance of survival. This is particularly crucial for stimuli providing socially relevant information for communication and interaction, such as faces. While it has been consistently demonstrated that the human brain shows preferential and ontogenetically early face-evoked activity, it is unknown whether specialized neural routes are engaged by face-predictive activity early in life. In this study, we recorded high-density electrophysiological (ERP) activity in adults and 9- and 4-month-old infants undergoing an audio-visual paradigm purposely designed to predict the appearance of faces or objects starting from congruent auditory cues (i.e., human voice vs nonhuman sounds). Contingent negative variation or CNV was measured to investigate anticipatory activity as a reliable marker of stimulus expectancy even in the absence of explicit motor demand. The results suggest that CNV can also be reliably elicited in the youngest group of 4-month-old infants, providing further evidence that expectation-related anticipatory activity is an intrinsic, early property of the human cortex. Crucially, the findings also indicate that the predictive information provided by the cue (i.e., human voice vs nonhuman sounds) turns into the recruitment of different anticipatory neural dynamics for faces and objects.

## Introduction

If someone knocks on our door, even if have no idea about who this person may be and what she or he may want from us, we can nevertheless rely on a minimum amount of a priori predictive information: we know that a person (rather than a pet) is standing behind the door. Consistent with this, our attention will be proactively biased toward preparing for social interaction. Conversely, if we hear the whistle of the teapot we know that the tea is ready and we therefore prepare to interact with an object, which intuitively does not engage us in social interaction.

These trivial examples clearly illustrate that our behaviour is shaped not only in response to external events but also by internal biases driven by one’s own past experience that allow us to generate predictions about the world’s facts. An interesting aspect consists of the fact that, beyond everyone’s lifespan experience (e.g., what to expect in a certain social situation and how to behave consequently), the ability to generate prediction can build on much simpler contextual experiences. For example, it is known that even short regular sensory sequences can induce statistical learning after just a few trials. This can be readily seen, for instance, in the surprise-related neural responses observed when an implicitly generated expectation is violated by incoming sensory evidence and, consequently, updated to incorporate new information^1^. This empirical evidence has been recently reframed by assuming that the brain is essentially a probabilistic predictive engine dedicated to minimizing the disparity between how it predicts the world to be and how it truly is^2,3^. While the phylogenetic importance of predictive cognition is undoubted, it is less known how this emerges ontogenetically across early human development. Decades of research clearly demonstrated that since birth, we are able to exploit environmental regularities and associate specific cues to determinate sensory events to build up an internal, statistical representation of the world that, in turn, proactively biases our cognition and behaviour^4^. The mismatch between the information stored in memory (our internal model of the world) and the actual incoming sensory inputs yields specific markers associated with expectancy violation (i.e., error prediction) at both the behavioural^5,6^ and neural level^7–10^. Notwithstanding this, a thorough developmental perspective that may offer a dynamic viewpoint about the emergence of predictive cognition from birth onward is still lacking. Indeed, while we know that the ability to both *generate* and *update* an internal model of the regularities of the world is an ontogenetically early property of the human brain^7–9^, less is known about the ability of young infants to *implement* predictive knowledge toward upcoming events to prepare for interacting with them in the most appropriate way. However, this aspect is far from trivial since one of the supposed phylogenetic functions of the predictive brain should be to ensure allostasis of the organism through error prediction minimization to optimize action^3^. In this light, a core scientific question is whether the ability to translate implicit predictive knowledge into the implementation of anticipatory neural activity follows developmental changes.

In a previous study, we used a Peekaboo simulation to induce temporal prediction of female faces in 9-month-old typically developing infants^11^. The findings identified, for the first time, that entraining young infants to temporally regular audio-visual stimuli induce a consistent neural anticipatory activity in the form a Contingent Negative Variation (CNV), a sustained, slow event-related potential (ERP) elicited during anticipation of sensory events even in the absence of overt behaviour^12,13^. In our study, we found that the CNV was underpinned by a distributed neural network engaged before stimulus presentation, implying that the translation of predictive cognition into anticipatory neural activity is an intrinsic property of the human brain that is established early in life.

However, since in the above study only human faces were used, an unsolved yet crucial issue is whether prediction-dependent neural anticipatory activity is a general, stimulus-independent brain property or, alternatively, shows category-specific features. More specifically, in the present study, we asked whether the associative learning of regular multisensory sequences (i.e., a sequence of an auditory stimulus followed by a visual stimulus) can induce different spatiotemporal anticipatory neural routes for socially relevant (faces) vs. irrelevant (objects) visual stimuli in preverbal infants. Previous studies identified specific electrophysiological markers for face processing as early as 3 months of age^14,15^, while a more recent investigation used EEG frequency tagging to show a partially right-lateralized network comprising lateral occipitotemporal and medial parietal areas overlapping with the adult face-processing circuit^16^. A neuroimaging study further showed evidence of a specific neural network in response to faces even earlier^17^. Remarkably, in this study, the neural pattern observed largely overlapped the adult face-processing network in 2-month-old infants, with core activation of the fusiform face areas. Furthermore, another important issue implied in the development of predictive cognition is the ability to understand the direct referential relationship between words and the entities they stand for. Marno et al.^18^ presented evidence that infants would have a basic expectation about the symbolic nature of verbal labels and that they would know that words can directly represent other entities (e.g., objects) in the world. The authors concluded that the early sensitivity to the characteristics of language demonstrated by human infants indicates an expectation about the referentiality of language, showing that infants as early as 4 months look for potential referents in their environment only when they hear somebody talking but not when the auditory signal lacks the characteristics of human speech (i.e., backward speech or no speech at all) along with an object-directed gaze of the speaker.

Taken together, the above mentioned evidence that (1) preverbal infants show expectancy-related anticipatory neural activity, (2) dedicated neural circuits for face processing are established early in life and (3) linguistic cues are required to elicit the referential expectation of the infant, opens the possibility that predictive-based neural activity may be stimulus-specific, depending on the human (i.e., voice) or nonhuman (e.g., jingles sounds) cue, with distinct spatiotemporal patterns forecasting distinct perceptual categories early in life. Following this research question, in the present study, we explored the anticipatory neural activity of 4- and 9-month-old infants and adults while waiting for being presented with social (human female faces) or nonsocial (objects) stimuli that were validly predicted by congruent auditory cues. These two age groups were chosen for the following reasons. First, we wanted to replicate extant findings that infants as young as 9 months can show reliable anticipatory brain activity^11^. Second, we aimed to extend previous findings to an earlier temporal window that is crucial for the development of sociocognitive and motor domains. The sophistication of a predictive brain, as well as emerging triadic social competences^19^, including referential expectations, is in fact expected to impact how 4- and 9-month-olds perceive and respond to others compared to general objects. Specifically, we expected (1) to observe prediction-based neural anticipatory activity in all groups, including the youngest one of 4 months, with a larger CNV for socially relevant (i.e., faces) than irrelevant (i.e., objects) stimuli and (2) to track developmental changes in the cortical routes engaged by social vs. nonsocial anticipatory activity. More specifically, we hypothesized that the neural generators engaged during anticipation may depend on the age-dependent level of experience with the expected object. In this light, on the basis of previous findings showing domain-relevant neural activity elicited *in response to* faces^14,17,20^, we assumed that both 9- and 4-month-old infants may exhibit an anticipatory recruitment of occipitotemporal areas *in anticipation of* faces. More specifically, in line with the assumption that face anticipatory activity mirrors the face-perception cortical pattern, we expect to observe a less distributed network of areas at 9 than 4 months of age due to the cortical specialization occurring during the first year of age^21,22^.

**Figure 1:**
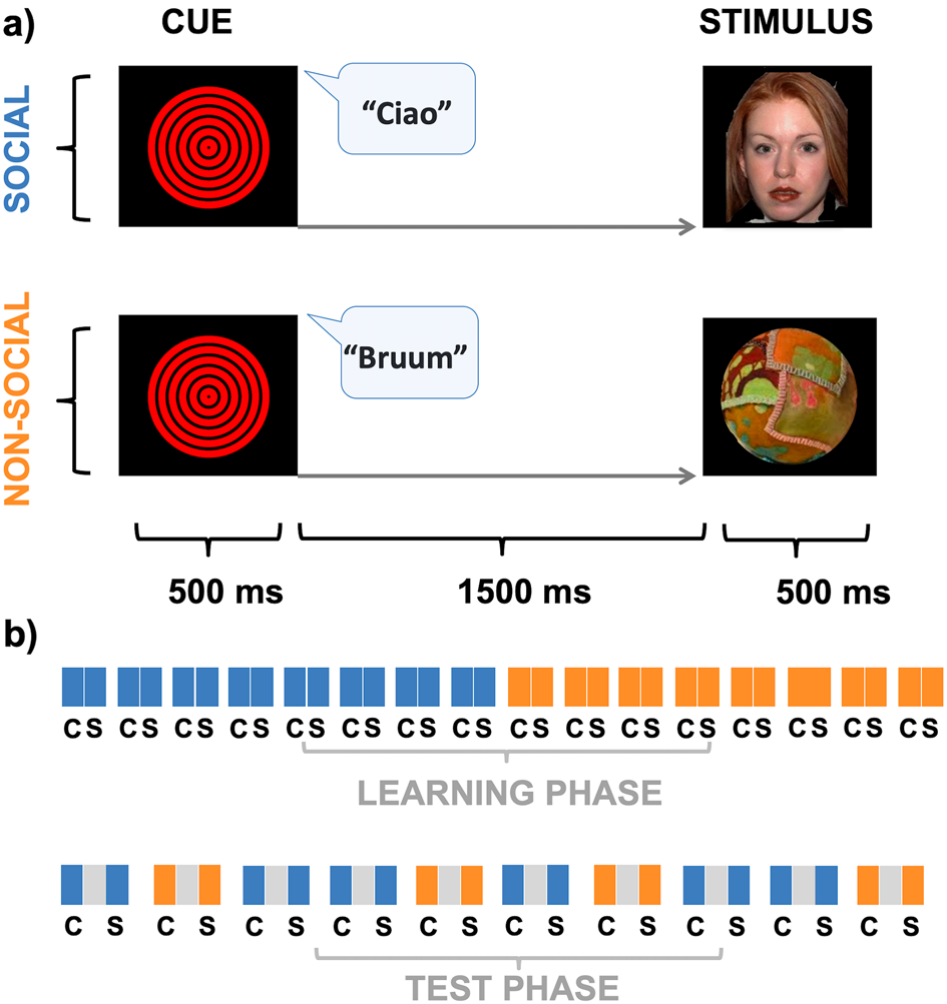
Experimental task (**a**). Both adults and infants are passively presented with an audiovisual sequence of stimuli including a cue (C) and a stimulus (S). The cue consisted of the synchronized presentation of a central “bull’s eye” acting as visual attention getter together with an auditory stimulus. According to experimental conditions, this could be a human female voice speaking Italian words (e.g.,/ciao/) or an inanimate object sound (e.g.,/bruum/), hence creating a social or nonsocial predictive context. After a fixed interval of 1500 ms, the cue is always followed by the display of a central picture on the monitor, which can be either an emotionally neutral, female face (social condition) or a rag ball (nonsocial condition). The cue-stimulus association is kept constant throughout the whole task, with human voices always predicting faces and sounds always predicting objects. Experimental design (**b**). At the beginning of the experiment, each participant underwent a learning phase in which the C-S sequence was presented sequentially and seamlessly eight times per category to induce associative learning between social and nonsocial items. After this, in the test phase, a fixed 1500 ms interval is embedded in the C-S sequence to elicit anticipatory neural activity before the onset of the stimulus.

To pursue this aim, both adult and infant participants were presented with a fixed sequence of items consisting of an auditory stimulus (cue) followed by a picture (stimulus). In one case, the sequence included a word spoken by a human female voice predicting the display of a human female face. We chose the human voice, as this kind of stimulus triggers infants’ attention very early. Indeed, even newborns are sensitive to the prosody, stress, rhythm, and intonation of the human voice due to their prenatal experience with low-filter action of the maternal uterine wall (300–400 Hz), which loses phonetic details but leaves unaltered prosody, melody, and rhythm of languages^23^. In a second control condition, the sequence included a nonhuman sound-like cartoon jingles or car sounds that was always associated with the following display of objects (Fig. 1).

We used a fixed interstimulus interval (ISI) to induce a fixed implicit temporal expectation and control for temporal uncertainty of stimulus onset. The fixed cue-stimulus association in the social (voices predicting faces) and nonsocial (sounds predicting objects) conditions was intended to allow participants to generate an internal model of sensory contingency. We assumed that implicit cue-stimulus associative learning should build up predictive knowledge about what to expect to see on the basis of the specific cue content (i.e., human voice vs. nonhuman sounds). We expected this prediction to in turn translate into a modulation of both anticipatory and stimulus-driven neural activity. To address this hypothesis, we collected high-density electroencephalographic activity (HD-EEG) and targeted both pre and poststimulus ERP activity associated with social (i.e., faces) and nonsocial (i.e., objects) pictures. We also exploited the optimal compromise between spatial and temporal resolution provided by the HD-EEG to estimate the ERP cortical generators with a source localization model based on infants’ realistic anatomical structure and electrical properties^24^. In line with the previous study of Mento and Valenza^11^, we hypothesized that the fixed cue-stimulus temporal contingency would overall turn into the allocation of general anticipatory neural resources, regardless of the social or nonsocial category of the stimulus expected. Specifically, in both adults and 9-month-old infants, we expected to replicate previous findings by observing sustained neural activity arising after the extinction of early cue-driven auditory ERPs and lasting for the whole ISI duration. Furthermore, in line with the hypothesis that anticipatory neural activity is an ontogenetically early intrinsic cortical property, we expect to extend previous findings to an earlier age by showing sustained prestimulus ERP activity in 4-month-old infants. The evidence of a sustained anticipatory neural pattern extending beyond the cue encoding window (i.e., > 1000 m s) in all groups would confirm that the temporal cue-stimulus association prompts the brain to generate temporal expectancy to actively prepare for upcoming inputs. In contrast, if participants are unable to learn the cue-stimulus temporal properties, we should expect the ERP activity to return to the baseline after the extinction of ERP activity evoked by cue processing (i.e., < 1000 ms).

In addition, we expected to go a step forward from the original observation of general anticipatory activity by showing that the infant brain is also able to build up category-specific anticipatory activity. Specifically, in line with the hypothesis that the developing brain is predictive in nature, we expected to find that the prediction of social vs. nonsocial stimuli would turn into different anticipatory neural patterns. Stemming from previous findings^17^, we hypothesize that in addition to eliciting a typical pattern of occipitotemporal neural responses, infants as young as 4 months old will show a similar activity while expecting the predicted presentation of a face.

## Results

### Prestimulus ERP activity

**Figure 2:**
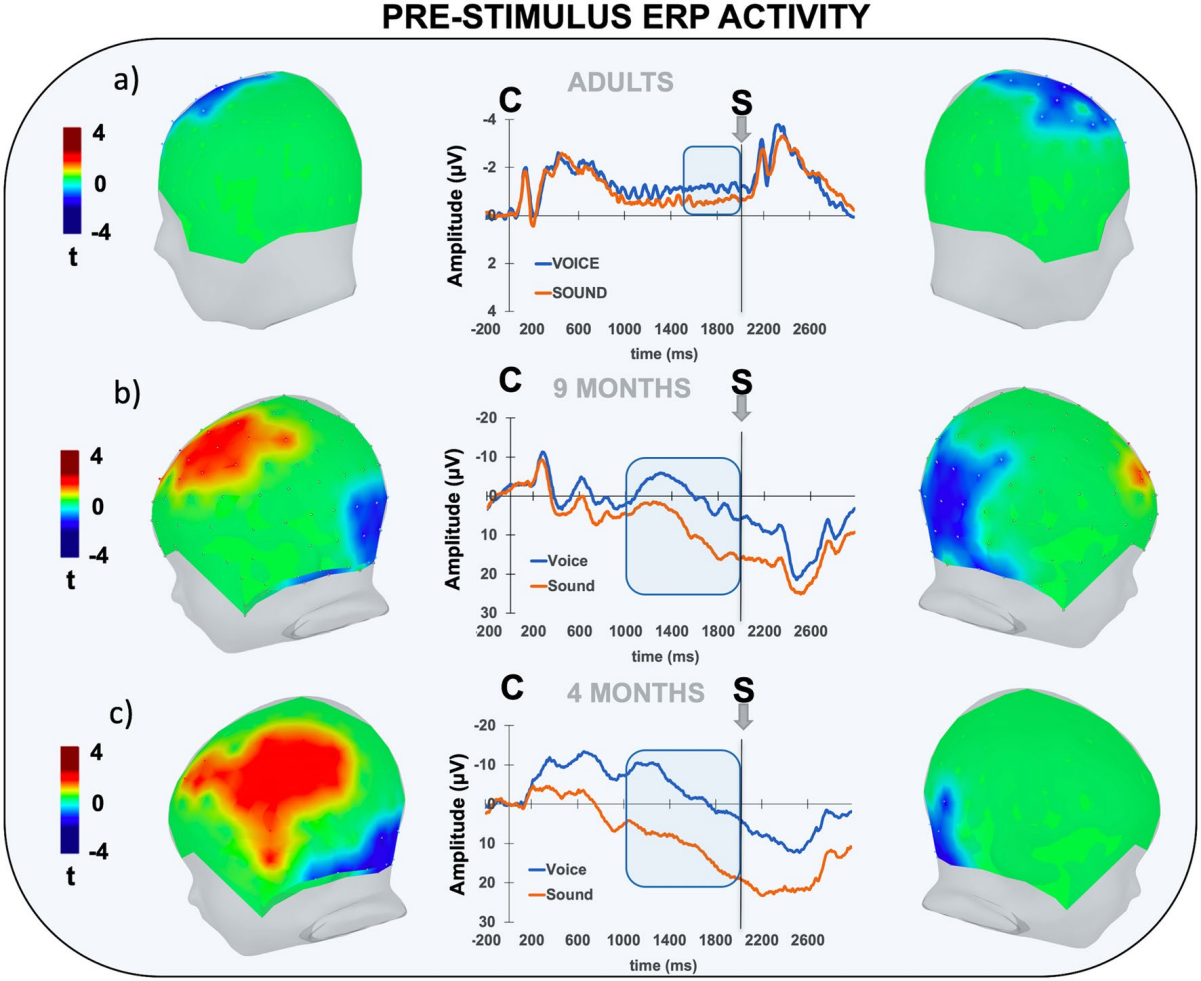
Prestimulus ERP activity. The picture shows the electrophysiological activity between the auditory cue (C) and the visual stimulus (S) separately for adult (**a**), 9-month-old (**b**) and 4-month-old (**c**) participants relative to the human voice (blue) and nonhuman sound (orange) cues. The plots of the t-score scalp maps show the spatial distribution (both left and right view) of the electrodes exceeding the critical t-score threshold for statistical significance at the cluster-based permutation test. In all groups, significant electrodes are grouped into distinct spatiotemporal clusters showing positive (in red) or negative (in blue) amplitude differences. The waveforms represent the grand-average ERP time course for each cluster. The blue bar on the x-axis represents the temporal extent of statistical significance for each cluster.

The visual inspection of the prestimulus neural activity revealed in all groups a clear electrophysiological pattern consisting of an early, cue-locked ERP complex including positive and negative transient voltage deflections. This complex reflected both sensory and cognitive processes underlying cue-encoding and extended over approximately one second. Then, instead of returning to the baseline, the ERP activity turned into a slow, sustained voltage amplitude that continued well beyond the cue-encoding time window (1000 ms), until visual stimulus presentation at 2000 ms from cue onset (Fig. 2). Consistent with previous studies investigating anticipatory ERP activity during passive viewing tasks, this late ERP activity was identified as the CNV^12^, a well-known neural marker of stimulus anticipatory activity^25^. Previous studies showed that the CNV is a slow ERP extending well beyond the early sensory-evoked neural activity and reflecting stimulus anticipatory activity even in the absence of explicit motor demand. For this reason, it can be considered a reliable hallmark of stimulus expectancy in preverbal infants^11,13,26^. Here, we observed that the CNV was present in both adults and 9-month-old infants, replicating previous findings^11^. Most importantly, we also found anticipatory neural activity in the youngest group of 4-month-old infants, demonstrating that stimulus expectation turns into anticipatory ERP activity at a younger age than previously reported^26^. Notably, we observed that the morphology and spatiotemporal features of the CNV were different between adults and infants. In fact, while in adults, the CNV showed the classical frontocentral distribution and negative polarity^25^, in infants, the CNV was spatially expressed as an anterior-positive/posterior-negative dipole. Moreover, we also observed that in adults, the CNV showed a stable negative polarity, while in infants, it declined below the isoelectric range, turning into positive absolute voltage values. These topographical and morphological differences have already been reported in previous CNV studies with infants and children^11,27^ and may be reasonably explained by a developmental difference in the neural generators underlying stimulus anticipatory activity. Despite this, in all groups, the CNV was greater (more negative) following human voice vs. nonhuman sound cues. Specifically, in adults, this effect was circumscribed over a cluster of centrally distributed electrodes that exhibited significantly greater negative amplitude upon presentation of voices than sounds (t >  ± 2.14; p_corr_ < 0.05; Cohen’s d = 0.3; Fig. 2a). This effect started at approximately 1500 ms following cue presentation and continued until visual stimulus presentation. We found the same effect at 9 months (t >  ± 2.14; p_corr_ < 0.05; Cohen’s d = 0.91; Fig. 2b), although in this case, it was expressed as a modulation of an anterior positive and a posterior negative cluster, with the latter being slightly right-lateralized. Finally, in the group of 4-month-old infants, we also found greater anticipatory activity following human voices than nonhuman sounds (t >  ± 2.14; p_corr_ < 0.05; Cohen’s d = 0.98; Fig. 2c). For the 9-month group, the CNV modulation was expressed as greater positive left frontal-central activation together with greater bilaterally distributed posterior negative amplitude. In both infant groups, the CNV modulation began at approximately 1000 ms posture, revealing an earlier effect than that found in adults.

### Post-stimulus ERP activity

**Figure 3:**
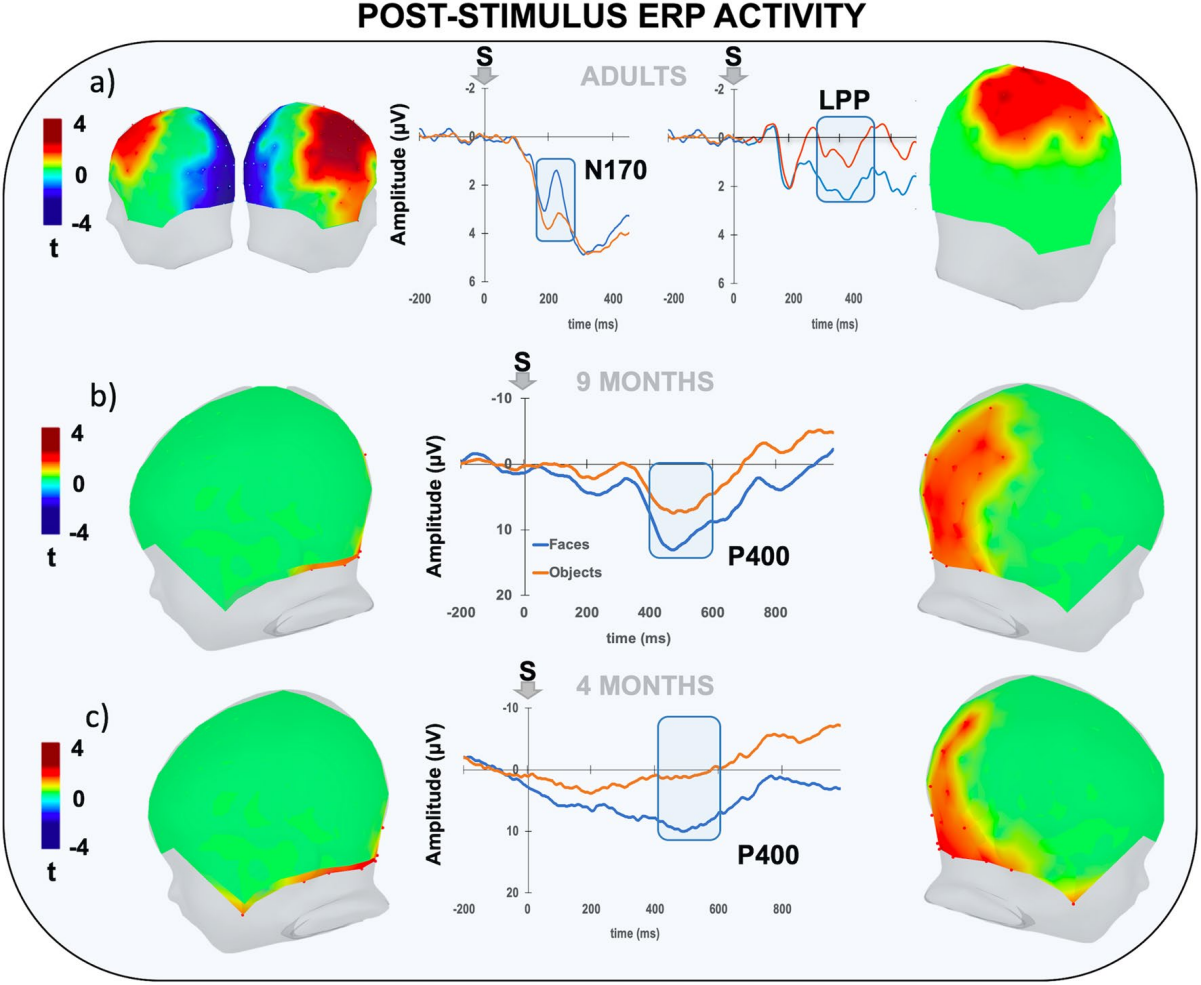
Poststimulus ERP activity. The picture shows the electrophysiological activity elicited by the visual stimulus (S) separately for adult (**a**), 9-month-old (**b**) and 4-month-old (**c**) participants relative to the faces (blue) and objects (orange). The lateral plots of the t-score scalp maps show the spatial distribution (both left and right view) of the electrodes exceeding the critical t-score threshold for statistical significance at the cluster-based permutation test. In all groups, significant electrodes are grouped into distinct spatiotemporal clusters showing positive (in red) or negative (in blue) amplitude differences. The waveforms represent the grand-average ERP time course for each cluster. The blue bar on the x-axis represents the temporal extent of statistical significance for each cluster.

The statistical analysis of the poststimulus ERP activity revealed the presence of significant effects in all groups. Specifically, the presentation of faces elicited a larger N170 amplitude in adults (Fig. 3a). This effect extended between 160 and 240 ms (mean peak maxima amplitude at 196 ms) from stimulus onset and was classically expressed in the bilateral posterior electrodes but also involved a cluster of anterior electrodes that showed more positive amplitude (t >  ± 2.14; p_corr_ < 0.01; Cohen’s d = 0.81). The N170 was followed by a centrally distributed late positive potential (LPP) initiating at approximately 300 ms from stimulus onset and lasting for several hundred milliseconds. As expected on the basis of previous studies, the LPP was significantly larger following faces than objects (t >  ± 2.14; p_corr_ < 0.01; Cohen’s d = 0.7). Indeed, this component has been suggested to reflect a variety of late mechanisms, including stimulus evaluation and controlled attention processes^28^, sustained attention^29^, motivational^30^, and attentional resources^31^ toward task-relevant or salient stimuli. Moreover, recent studies further demonstrated that LPP is sensitive to the degree of expectancy of the upcoming stimulus^32,33^. In line with this, the fact that in adults we found a larger LPP for highly expected faces rather than objects is not surprising.

In the 9-month-old babies, the onset of faces elicited the classical posterior negative N290, although this component was not significantly affected by stimulus condition in either 9- or 4-month-old infants. A larger P400 amplitude following faces (than objects) over a right posterior cluster of electrodes was also found (Fig. 3b). This effect extended between 400 and 550 ms (mean peak maximum amplitude at 440 ms) from stimulus onset (t >  ± 2.14; p_corr_ < 0.01; Cohen’s d = 1.2). Likewise, in the 4-month-old babies, faces elicited larger P400 amplitudes than objects at the right posterior cluster of electrodes (Fig. 3c). As expected, probably due to maturational factors, this effect was slightly later than what was observed at 9 months since it extended between 400 and 700 ms (mean peak maxima amplitude at 480 ms) from stimulus onset (t >  ± 2.14; p_corr_ < 0.05; Cohen’s d = 1.1).

### Correlations between pre and poststimulus neural activity

**Figure 4:**
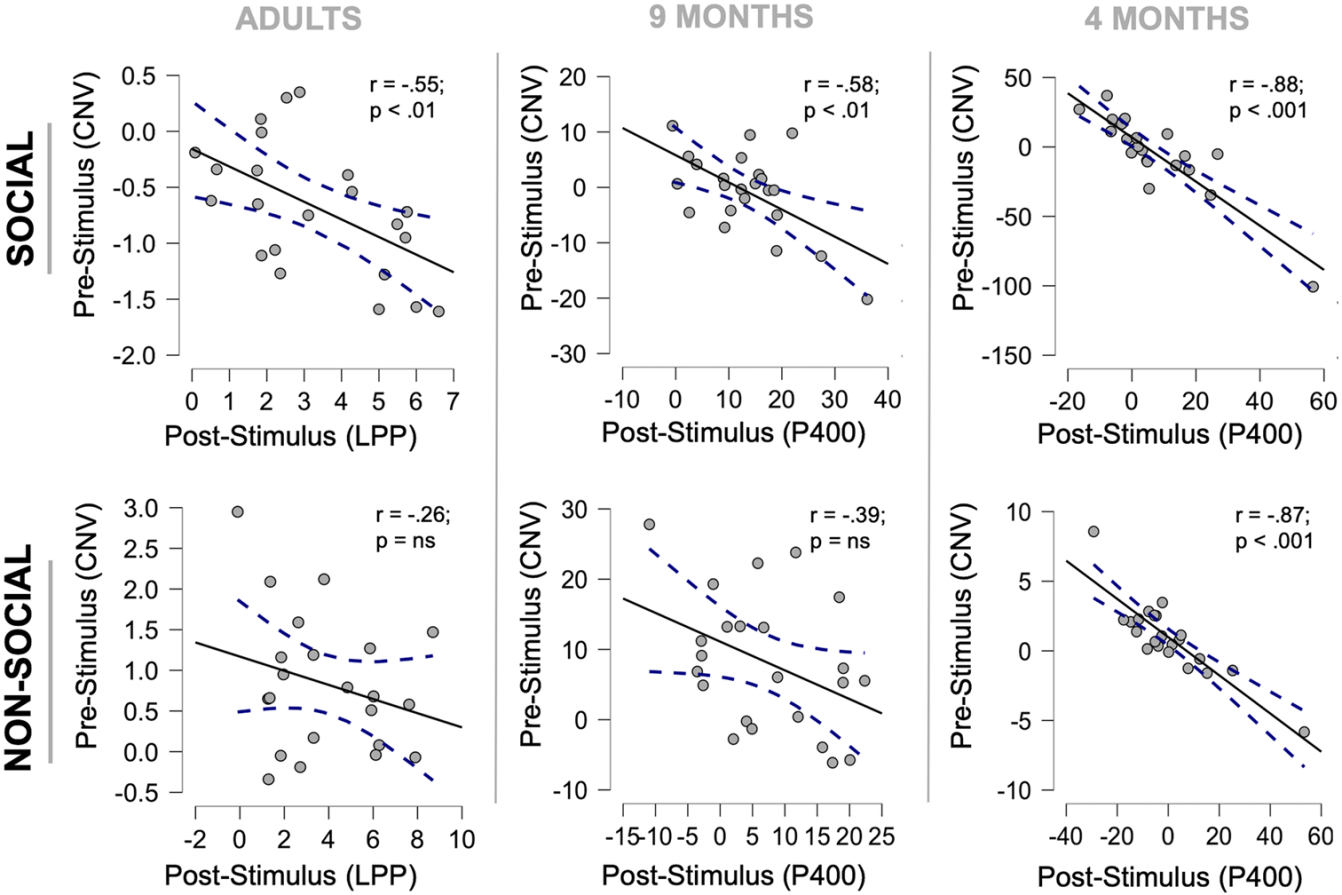
Correlation between pre and poststimulus ERP activity. The plots show the single-subject poststimulus ERP activity (x-axis) as a function of prestimulus ERP amplitude for social (top) and nonsocial (bottom) conditions (y-axis) for adults (left column), 9-month-old (central column) and 4-month-old infants (right column). The dotted lines represent the confidence intervals.

The correlations between pre and poststimulus neural activity are reported in Fig. 4, separately per group and conditions. For each group, we targeted the components whose amplitude was significantly modulated by condition. Specifically, for the prestimulus window, we targeted the CNV in all groups. Regarding the poststimulus activity, we targeted the N170/LPP and the P400 as neural markers for adults and infants, respectively. For each component, we averaged the voltage values over the temporal interval and within the cluster of electrodes showing significant modulations from the mass univariate analysis.

Notably, we found that, relative to the social condition, the prestimulus activity predicted the poststimulus ERP amplitude in all groups. That is, the more negative the CNV induced by human voices was, the larger the positive ERP responses (LPP for adults and P400 for infants) to face onset. No significant correlations were found for the N170. This correlation was significant in all groups (adults’ r = − 0.55; p < 0.01; 9 months’ r = − 0.58) but reached the greatest magnitude in the 4-month group (r = − 0.88; p < 0.001). Concerning the nonsocial condition, we found no significant pre-post stimulus correlation for either adults (r = − 0.26; p = ns) or 9-month-old infants (r = 0.39; p = ns), while at 4 months, this correlation was equally strong as in the social condition (r = − 0.87; p < 0.001). Overall, these findings indicate that the anticipatory activity (CNV) induced by social cues (human voices) predicts the amount of neural resources allocated to process face stimuli both in adulthood (LPP) and in infancy (P400). In contrast, anticipatory activity was equally predictive for social and nonsocial stimuli only in the youngest group of 4-month infants.

### Brain source reconstruction

#### Social vs. nonsocial source activity

**Figure 5:**
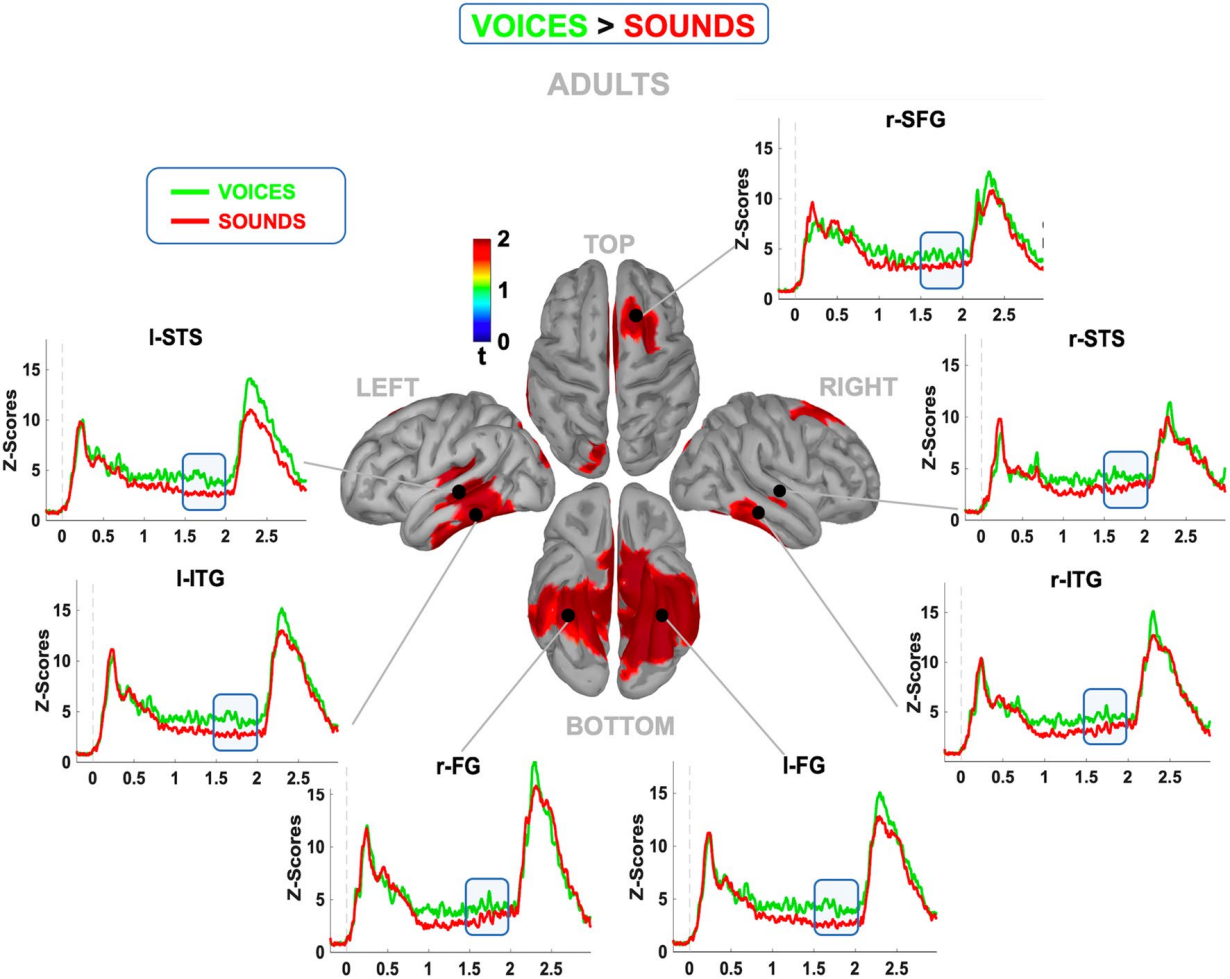
Prestimulus cortical source reconstruction (voices vs. sound) in adults. The maps display the reconstruction of the averaged cortical activity relative to the portion of CNV significantly modulated by the cue (blue bar) in adults. The surrounding panels show the comparison between the time course of the averaged normalized and smoothed activity induced by human voices (green line) vs. nonhuman sounds (red line) relative to the cortical regions showing significant modulation (p < 0.05; uncorrected).

The brain source reconstruction revealed a complex pattern of distributed cortical regions in all groups whose activity was significantly modulated by cue type during the CNV time window. Specifically, when contrasting the prestimulus reconstructed source map between social and nonsocial conditions, we found a cluster of face expectancy-sensitive areas that partially overlapped and were partially different in the three groups. Specifically, as shown in Fig. 5, in adults, we identified a group of regions including frontal (the right superior frontal gyrus or r-SFG) as well as temporal dorsal (inferior temporal gyrus or ITG and superior temporal sulcus or STS, bilaterally) and ventral (fusiform gyrus or FG, bilaterally) areas showing higher functional activity before faces than objects (p < 0.05, uncorrected). The visual inspection of the time course clearly revealed that all single areas showed a stronger activity at least 500 ms before face onset, suggesting that they are part of a distributed, category-specific network engaged before predictable stimuli.

**Figure 6:**
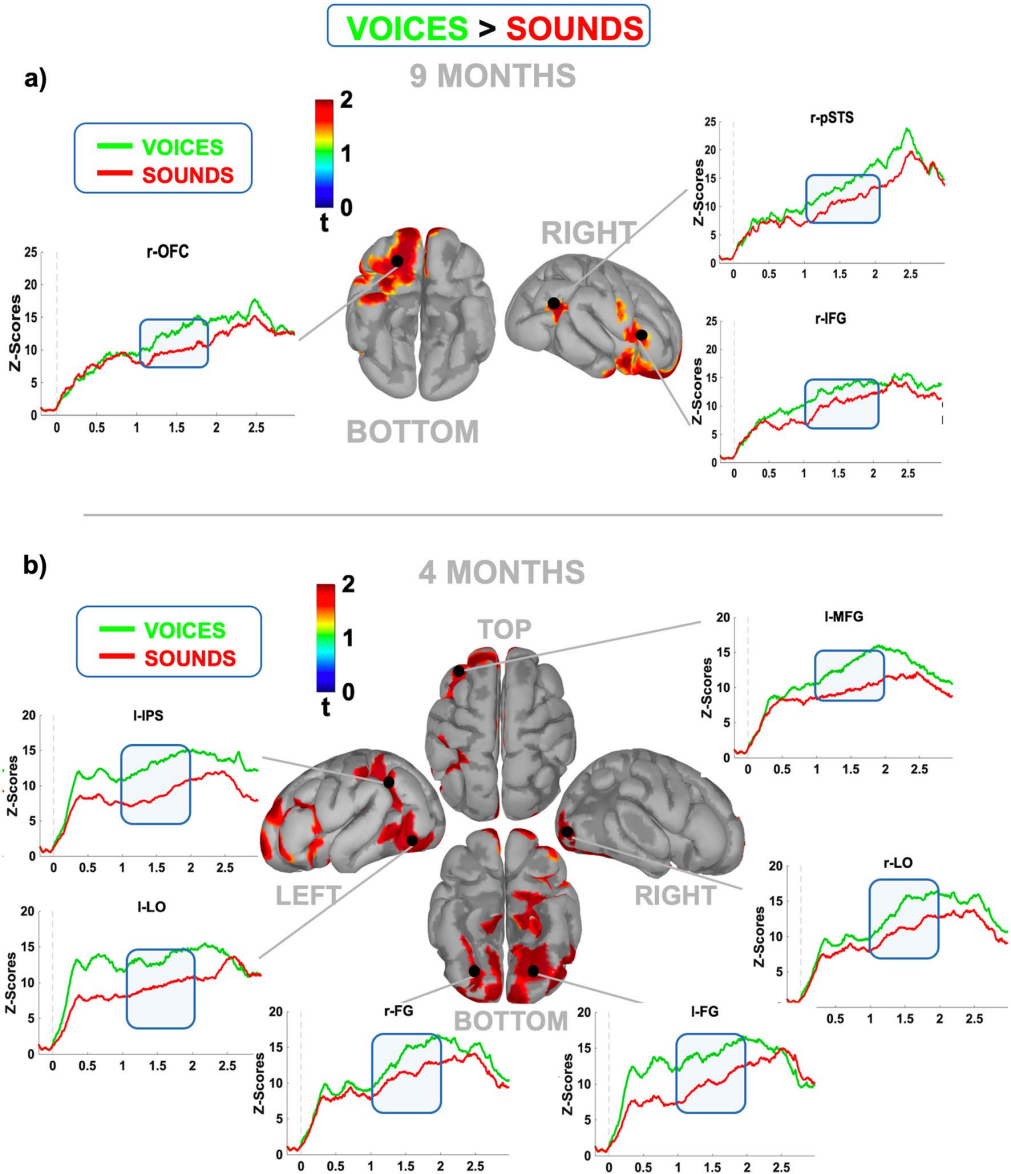
Prestimulus cortical source reconstruction (voices vs. sound) in infants. The maps display the reconstruction of the averaged cortical activity relative to the portion of CNV significantly modulated by the cue (blue bar) in 9- (**a**) and 4-month-old (**b**) infants. The surrounding panels show the comparison between the time course of the averaged normalized and smoothed activity induced by human voices (green line) vs. nonhuman sounds (red line) relative to the cortical regions showing significant modulation (p < 0.05; uncorrected).

In the 9-month-old group, we also found a consistent pattern of areas that showed a significant cue-dependent modulation in the CNV time range (p < 0.05, uncorrected). As shown in Fig. 6a, two distinct frontal regions (right orbitofrontal cortex or r-OFC and right inferior frontal gyrus or r-IFG) and a temporal dorsal region (right posterior superior temporal sulcus or r-pSTS) were selectively activated during face expectation. Finally, in 4-month-old babies, a more distributed cortical pattern was engaged during face expectancy, including temporal ventral (FG) and occipital (lateral occipital or LO cortex) gyrus bilaterally as well as left parietal (left parietal temporal sulcus or l-IPS) and prefrontal (l-MFG) areas (Fig. 6b).

#### Nonsocial vs. social source activity

**Figure. 7:**
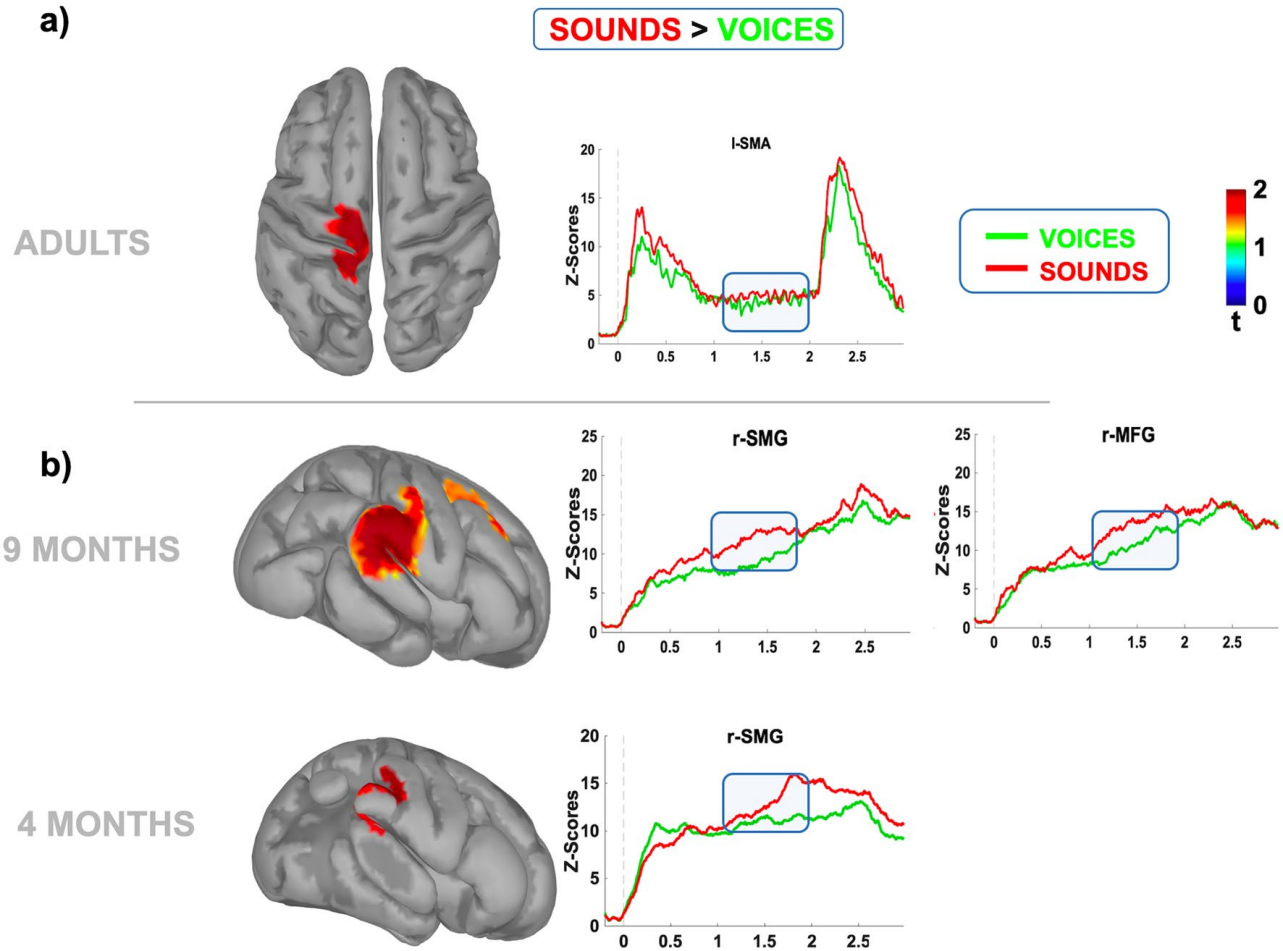
Prestimulus cortical source reconstruction (sound vs. faces) in adults and infants. The maps display the reconstruction of the averaged cortical activity relative to the portion of CNV significantly modulated by the cue (blue bar) in adults (**a**) and infants (**b**). The right panels show the comparison between the time course of the averaged normalized and smoothed activity induced by human voices (green line) vs. nonhuman sounds (red line) relative to the cortical regions showing significant modulation (p < 0.05; uncorrected).

When contrasting the prestimulus source activity prompted by nonhuman sounds vs. human voices, we found a spatially focused, age-dependent neural pattern in all groups (Fig. 7). Indeed, we found that the expectancy of an object was circumscribed over the central cortical regions in adults, engaging the left precentral (l-PC) areas and, partially, the left supplementary motor area (l-SMA). However, in both 4- and 9-month-old infants, we found that the right supramarginal gyrus (r-SMG) was strongly preactivated before objects. Remarkably, only at 9 months of age did we report an additional recruitment of frontal activity with the involvement of the right middle frontal gyrus (r-MFG).

## Discussion

In the present study, we provide, for the first time, developmental evidence of early anticipatory neural activity induced by predictive information of either socially relevant (faces) or irrelevant (objects) stimuli. We entrained both adults and preverbal infants of 9 and 4 months to associate, in one case, social auditory cues (a word spoken by a human female voice) with social visual stimuli (female faces). In another condition, they learned to associate nonsocial auditory cues (nonhuman sounds) with pictures of nonsocial objects (rag balls). This association was always valid, and the two conditions were randomly alternated trial-by-trial. As a first result, we replicated previous studies showing that a temporally regular cue-stimulus association induces neural anticipatory activity in both adults^12^ and 9-month-old infants^11,26^ even in the absence of overt behaviour. Indeed, we report clear evidence of sustained, anticipatory ERP activity in the form of a frontocentral CNV arising early soon after the end of the cue-locked auditory evoked responses and continuing until stimulus onset. Remarkably, here we move back the developmental onset of anticipatory neural processes, showing for the first time to our knowledge that the CNV can also be reliably elicited in infants as young as 4-months. In this way we provide further evidence that expectation-related anticipatory activity (i.e., prediction implementation stage) is an intrinsic, early property of the human cortex, as previously proposed for updating elicited by expectancy violation^7^.

In addition, our findings step beyond previous evidence by showing that, rather than being general, predictive processes operate through category-specific anticipatory neural mechanisms both in adults and infants, engaging distinct neural correlates according to the specific perceptual content of predictions. This was clearly indicated by larger CNV amplitudes before face than object presentation, supporting the interpretation that the possibility of generating a prediction of incoming inputs potentially relevant for social interaction engages stimulus category-specific neural resources. Remarkably, a larger recruitment of anticipatory neural activity predicts a greater allocation of computational resources on stimulus processing, although this held only for social stimuli. Indeed, the larger the CNV was before face processing, the larger the amplitude of the face-sensitive ERP markers in all groups. Specifically, in the social condition, the CNV predicted the LPP amplitude in adults, as it did in infants for the face-sensitive P400 amplitude. In contrast, the CNV elicited by nonsocial cues was not significantly related to object-locked ERP activity, except for the youngest group, which showed a very large cue-stimulus ERP correlation for both categories.

Furthermore, while the CNV exhibited similar temporal dynamics, its category-specific modulation presented different scalp distributions in adults compared to infants. In fact, while in adults the CNV modulation was neatly localized over central scalp electrodes, in infants this effect exhibited a dipolar configuration, with a posterior negative/anterior positive distribution. These dissimilar spatial patterns indicate different underlying neural generators, suggesting that anticipatory neural activity induced by predictive cues goes through ontogenetic changes from infancy to adulthood. Moreover, we observed a further scalp distribution difference between 4- and 9-month-old infants, suggesting that at least partially different neural generators support predictive mechanisms across early development. To follow up on this finding, we applied a source reconstruction of the CNV modulatory effect by using age-appropriate templates. Overall, in all groups, we found higher preactivation of a pool of cortical areas traditionally associated with face processing. These included occipitotemporal areas such as the superior temporal sulcus, the inferior temporal gyrus and the fusiform gyrus. Moreover, we also found that face prediction turned into the recruitment of parietal and prefrontal lobes, suggesting that face expectancy involves brain areas traditionally associated with both domain-specific and domain-general processes. It is worth noting that all these areas exhibited enhanced anticipatory activity in a time window extending well beyond the sensory, perceptual and cognitive computational stage associated to cue encoding, suggesting that this neural signature is functionally related to the forecoming visual stimulus rather than merely reflecting the processing of the cue auditory features. In support of this, previous evidence clearly showed that social stimuli such as voices or faces elicit a neural stream reflected at scalp level by several ERP components occurring between about 100 and 1000 ms from stimulus onset. Among these, those occurring in an early window (i.e., from 50 to 500 ms) are expected to be obligatory and have traditionally been related to stimulus encoding including sensory and perception stages. By contrast, ERP components peaking between 500 to 1000 ms are sensitive to more demanding word- or face-related cognitive processing, including attention, memory and emotional processes engaged in stimulus meaning extraction^34^. In S1-S2 experimental paradigms ERP signal extending over this temporal window reflects representational neural activity most probably tracking anticipatory ERP activity (i.e., CNV) underlying stimulus preparatory processes. These can embody multiple predictive information that can effectively bias attention and shape behavior, including temporal expectation^35–37^ or perceptual features^38^. Consistently with adult literature here we report neural signatures of category-related predictive neural activity in preverbal infants.

The most remarkable finding was that this distributed network was not common in all groups, since age-related differences were present. In particular, 4-month-old infants showed a distributed pattern entailing the fusiform gyrus and the lateral occipital cortex bilaterally. No prestimulus middle or superior temporal lobe activations were found in this group. In contrast, in the 9-month-old group, the ventral occipitotemporal areas were not significantly recruited by face prediction, while we found selective involvement of the right posterior temporal sulcus (r-pSTS). Both ventral and dorsal temporal areas were activated before face onset in adults, although in this case, the portion of the temporal sulcus that was involved was more anterior than what was observed at 9 months. Taken together, the recruitment of the occipital and temporal ventral and dorsal routes induced by face expectancy is a remarkable finding since it suggests an ontogenetically early predisposition of the human brain not only for processing socially relevant stimuli^39^ but also for preparing to them. Importantly, to our knowledge, this is the first evidence that very young infants can prepare for socially relevant stimuli by recruiting face-sensitive areas hundreds of milliseconds before the actual presentation of a face. Remarkably, while both 4- and 9-month-old babies allocated face-specific anticipatory neural resources, we report here several developmental differences.

We believe that, if corroborated by further experimental evidence, this result may be remarkable since it may, on the one hand, shed a new developmental perspective on the way human beings build knowledge of the world in terms of social predictive cognition. On the other hand, it may provide a new avenue to investigate neurodevelopmental disorders characterized by a failure of social predictive processes, such as autism spectrum disorder^40,41^.

As a noticeable finding, when contrasting the brain activity preceding objects to the one preceding faces, we found a different pattern of anticipatory electrophysiological activity. Indeed, in all groups, we observed no ventral posterior activity over the occipitotemporal areas but, rather, an engagement of distinct areas according to age. Specifically, adults showed frontocentral activation spreading over the left supplementary motor area (SMA). The SMA has been traditionally associated with motor processing, including action planning, response setting, preparation and selection^42,43^. Yet, this area is also involved in the absence of explicit motor demands, as in the case of tasks inducing a covert, action-independent temporal expectancy^12,13^ This suggests that, in adults, action-related processing may to some extent underlie, or at least share, brain circuits with perceptual timing mechanisms^44–46^. Indeed, an accurate timing of forthcoming events is of crucial importance for limb-movement execution and, more generally, for motor preparation, even in the case in which an overt motor response is actually not required. It follows that t he involvement of the SMA during stimulus expectancy might be age-dependent, depending on both the motor repertoire and object-interaction experience that every participant developed across the lifespan. In line with this hypothesis, we found a developmental dissociation between 4- and 9-month-old infants, with only the latter showing a clear involvement of the superior frontal gyrus (incorporating the SMA) together with the right supra-marginal gyrus. In contrast, 4-month-old infants only exhibited object-anticipatory activity over the SMG. Taken together, these findings seem to suggest a developmental trend based on the common recruitment of the SMG in infants, spreading over more frontal areas only at the age of 9 months. While the role of SMG is still not completely clear at this age, it can be put forward to play a role in top-down orienting of visual attention toward an upcoming relevant event. However, only after determinate milestones in motor and social development have been reached does it make sense to expect an additional involvement of frontal areas potentially engaged in object interaction.

In support of this hypothesis, we should consider that while 4-month-old infants have experience of social interaction and exposure to faces from the very first instant of their life^47^, they do not yet experience intentional motor interaction with objects. In fact, their neurological maturation and motor repertoire do not allow them to intentionally interact with objects by implementing reaching or grasping actions, since this ability develops after 5 months of age^48^. In other words, they have not yet developed action affordance towards physical objects. In contrast, at 9 months of age, infants have already experienced intentional interaction with the physical word: they can voluntarily reach and grasp objects when they are in front of them^49^. Hence, it is reasonable that their motor system can operate not only in a reactive way but also proactively, as consistently reported for older children and adults^11,12^. The preactivation of anterior cortical areas at 9 months but not at 4 months may be therefore explained by the intentional interaction with objects emerging only after approximately 5 months of age. A further argument supporting the role of previous social experience can build on the linguistic nature of the auditory cue we used. Indeed, the development of social predictive cognition implies the ability to understand the direct referential relationship between words and the objects they stand for. Marno et al.^18^ provided evidence that infants would have a basic expectation about the symbolic nature of verbal labels and that they would know that words can directly represent other entities in the world. The authors suggested that the early responsiveness to linguistic stimuli shown by human infants could be considered as a sign of referentiality of language, demonstrating that infants as early as 4 months old look for potential referents in the surrounding environment only when they hear a human voice, but not when the auditory sound is lacking the characteristics of human speech (i.e., backward speech or no speech) along with a direct gaze at the speaker's object.

Finally, it is also noteworthy that behavioral and neuroimaging studies have demonstrated a close relationship between motor and language development, highlighting that the emergence of language skills involves not only the brain areas devoted to language but also to movement^50,51^. Overall, these studies highlight that age-related language development, in addition to motor skills refinement, contributes to changes in the way children act and interact with objects, further explaining the anticipatory involvement of motor areas at 9 compared to 4 months of age. However, disentangling the exact role of linguistic vs. motor experience in building the way the developing brain builds predictive-based anticipatory activity is beyond the original aim of the present study and deserves further investigation.

In conclusion, our findings suggest that our experience of the world not only constrains the way we process the stimuli after they physically occur but also the way we prepare to interact with them. We suggest that this predictive process might be functionally mediated by an interaction between biologically constrained, ontogenetically early mechanisms and experience-dependent, developmentally emerging changes, in line with a neurocostructive account of human cognitive development^52^.

From a theoretical perspective, the present study adds new knowledge supporting the account of the human brain as a complex machinery biologically predisposed to construct predictions about external events. More specifically, our findings might suggest an ontogenetic continuum between infants and adults in the mechanisms at the basis of expectation-based anticipatory behaviour. In fact, notwithstanding the bulk of structural and functional changes occurring during development, the capacity of the human brain to extrapolate environmental sensory regularities and proactively shape its cortical activity to the structure of the environment may constitute a basic, early property of the human neural architecture.

## Methods

### Participants

A total of seventy-five participants took part in the study, including twenty-three adults, thirty-one 9-month-old infants and thirty 4-month-old infants. Among these, sixteen infants were rejected because of fussiness, excessive movements or insufficient number of clean trials after EEG preprocessing. The final sample included twenty-three adults (mean age 23 ± 1 (SD) years; range 21–26; 19 females), twenty-three 9-month-old infants (mean age 287 ± 24 (SD) days; range 247–333; 15 females) and twenty-two 4-month-old infants (mean age 133 ± 13 (SD) days; range 119–167 days; 5 females). All adults participants, children's parents and/or their legal guardians signed an informed consent for both study participation and publication. All experimental methods had ethical approval from the Research Ethics Committee of the School of Psychology of the University of Padova, Italy (prot. N. 1179). The methods were carried out in accordance with the approved guidelines and regulations.

### Stimuli

All participants were presented with synchronized audio–visual sequences of stimuli. Auditory stimuli consisted of digitized human voices or nonhuman sounds. Human voices were sampled from volunteers and consisted of four Italian words of very common use, i.e.,/*ciao*/,/*ecco*/,/*guarda*/,/*pronti*/ (/hello/,/here/,/look/,/ready/), none of them referring to a specific referential target. Nonhuman sounds consisted of typical sounds of objects, including jingles, cartoon-like rings or car sounds. Visual stimuli consisted of pictures of 6 real, emotionally neutral, female faces or 6 coloured rag balls. The face stimuli were selected from the NimStim Set of Facial Expressions^53^, while the object stimuli consisted of publicly available pictures from the internet. All stimuli were matched by dimensions and low-level physical features such as luminosity and contrast. All infants seated on their parent’s lap at a viewing distance of about 60 cm from a 24-in. computer monitor (1280 × 1024 resolution). The maximum experiment duration was about 10 min considering the total number of trials.

### Experimental paradigm

The trial sequence and experimental design are shown in Fig. 1. Each trial began with the presentation of a first stimulus (cue), which could be either a voice or a nonhuman sound (equally and randomly delivered), for a duration of 500 ms. To maintain infants’ attention at the center of the monitor, the cue was always associated and synchronized to the presentation of a central “bull’s eye” fixation figure, characterized by six concentric red circles (450 × 338 pixels). After a fixed interstimulus interval (ISI) of 1500 ms, the cue was always followed by the presentation of a central picture on the screen (stimulus). This lasted 500 ms on the screen and could represent either a female face or an object. At the beginning of the experimental block, there was an initial, 16 -trial learning phase (i.e., 8 social and 8 non-social trials) in which no intervals were embedded between the cue and the stimulus. The learning condition order was randomized subject-wise so that a participant received first the social trials and then the non-social ones and the following one the opposite order.

This was done to induce the association between voice-face and sound-object. Then, the test phase was introduced, in which the cue and the stimulus were separated by a fixed 1500 ms ISI. The introduction of a constant cue-stimulus temporal delay allowed us to induce an exogenous temporal expectancy, consequently, to elicit anticipatory neural activity during the prestimulus interval^11,13^. On the other hand, this allowed us to investigate whether the predictive information furnished by the cue (i.e., social vs. nonsocial) could turn into the recruitment of different anticipatory neural dynamics for faces and objects. The adults underwent the whole experiment, which included a maximum of 200 trials (100 per condition type; randomly delivered). Differently, for infants the experiment continued as long as they were able to keep their visual attention on the monitor and was stopped as soon as they showed insensitivity to attentional catchers displayed on the screen. In any case, t he recording session was stopped if the infant became fussy or after the end of the experimental block. The intertrial interval (ITI) was randomly varied between 500 and 1500 ms.

### EEG recording and signal processing

The EEG was continuously recorded and amplified using a geodesic EEG system (EGI GES-300) through a precabled high-density 128-channel HydroCel Geodesic Sensor Net (HCGSN-128) and referenced to the vertex. The sampling rate was 500 Hz. The impedance was maintained below 60 kΩ for each sensor. All EEG recordings were processed offline using the MATLAB toolbox EEGLAB^54^ and BRAINSTORM software^55^. A bandpass filtered between 0.1 and 30 Hz was applied. During the whole session, the infants’ gaze was monitored via a camera. This allowed a trained experimenter to manually display on the screen audio-visual stimuli (cartoon scenes) to recapture infants’ attention on the screen whenever they looked away. Moreover, two trained researchers detected off-line the trials in which the visual stimuli were disregarded. Only trials obtaining a double rejection judgements were discarded from analyses. The agreement rate was > 90%. To depict the temporal dynamics of brain activity induced by different stimulus predictions, epochs between − 200 and 3000 ms from cue onset were extracted. This window included both pre and post-stimulus neural activity. Channels that exceeded a differential average amplitude of ± 300 (infants) or 100 (adults) μV on more than 30% of all epochs were marked as bad, excluded and subsequently interpolated with the spherical spline interpolation method^56,57^. Epochs having more than 20% (infants) or 10% (adults) bad channels were also excluded. A mean of 1.9 ± 1.2 (adults) and 6.4 ± 2.6 (infants). electrodes were considered bad and interpolated. Data were subjected to independent component analysis (ICA^58^) to isolate highly characteristic artifacts, including eyeblinks, movements and heartbeats. Artefactual components were discarded, and the remaining components were projected back to the electrode space to obtain cleaner EEG epochs. Data were then rereferenced to the average of all electrodes, and the signal was aligned to the baseline, which included 200 ms before cue and stimulus onset for both pre and poststimulus temporal windows. To rule out any potential bias due to prestimulus CNV amplitude differences on poststimulus ERPs we further considered two additional baseline correction methods. These included both a narrow (− 50 to 50 ms) and extended (− 1000 to 0 ms) correction windows centred on S2 onset. In both cases the ERP effects were statistically confirmed. For the sake of simplicity here we only report the − 200 to 0 ms baseline correction. Subject average and grand average ERPs were generated for each electrode site and cue type. For adults, a total of 90 ± 12 and 90 ± 11 clean trials were obtained for social and nonsocial conditions, respectively. For the 9-month group, a total of 30 ± 7 and 29 ± 7 clean trials were obtained for social and nonsocial conditions, respectively. For the 4-month group, a total of 31 ± 8 and 31 ± 7 clean trials were obtained for social and nonsocial conditions, respectively. No significant differences in the number of trials per condition were found within each group (all t_s_ < 1; all p_s_ > 0.7).

### Pre and post stimulus target time windows

To test for the presence of significant differences in the stimulus anticipatory neural activity elicited by social vs. nonsocial cues, in all groups, we compared the ERP amplitude in the temporal window between 1000 and 2000 ms following cue presentation. We chose this temporal window according to a previous study adopting a similar paradigm^11^ as well as after a careful visual inspection of the grand-averaged ERP waveforms. Indeed, in all groups, the ERP activity elicited by cue onset extended over a window of approximately 1000 ms and consisted of rapid and transient voltage changes. After this time, the ERP signal became slow and sustained, indicating the presence of prestimulus anticipatory activity. In the poststimulus activity, we targeted the window between 150 and 300 ms for the adult N170 component. For infants, we targeted both the N290 (200 to 400 ms) and P400 (300 to 700 ms) components. These windows were selected according to previous literature^14,20,59^.

### Statistical analysis

For each contrast, we applied a pairwise, cluster-based permutation test based on the cluster mass univariate statistic^60,61^ using the original data and 1,000 random within-participant permutations of the data. This method allows statistical testing with no need for a priori selection of regions of interest since it controls for multiple comparisons by clustering neighboring channel pairs that exhibit statistically significant effects. Electrodes within approximately 1.5 cm of one another were considered spatial neighbours, and adjacent time points were considered temporal neighbours. All pairs whose t values were larger than a predetermined threshold of ± 2.14 (corresponding to a familywise error corrected alpha value of 0.05) were considered significant. The p value of each statistically significant cluster is indicated as p_corr_ to mark that it is corrected for multiple comparisons.

### Correlations between pre and poststimulus neural activity

To directly test whether the neural activity elicited by the cue was predictive of the neural activity evoked by stimulus presentation, we extracted both pre and poststimulus mean ERP activity. Specifically, for each participant and condition (i.e., social vs. nonsocial), we extracted the mean voltage amplitude averaged across space (electrode clusters) and time (sampling points) showing statistically significant voltage modulations. This was done separately for each group, as different ages showed different spatiotemporal scalp effects. All the individual measures were extracted from either the postcue or the poststimulus windows and correlated with each other by means of a two-tailed Pearson’s correlation. This was done for each group to further address the question of whether the stimulus predictive activity is developmentally constrained.

### Brain source reconstruction

Although a direct comparison of brain source reconstruction across different ages is made difficult by the presence of possible brain structural differences, the use of a high-density electrode array (i.e., ≥ 128), together with other age-appropriate adjustments (see below), allowed us to cautiously attempt a qualitative comparison of the cortical areas generating anticipatory neural activity at different ages. To reconstruct cortical sources, we applied the same procedures described in the literature for adults^35^ and infants^9,11^. In particular, the cortical sources of the averaged ERP signal for each condition and participant were reconstructed using Brainstorm^55^. Age-appropriate templates for adults (MNI templates) and 9- and 4-month-old infants^24^ were used after warping the geometry of the EEG sensor net to fit the head mesh. This procedure considerably improved the spatial resolution of brain source maps since it allowed us to project functional activations on a structural MRI space closely approximating the real cortical surface anatomy at different ages. The solution space was constrained to the cerebral cortex, which was modeled as a three-dimensional grid of 15,002 fixed dipoles normally oriented to the cortical surface. The EEG forward solution was computed using a realistic model for adults^62^ and an overlapping 3-sphere model^63^ for infants since a realistic model is not yet publicly available for infants. Cortical current maps were computed from the EEG time series using the sLORETA algorithm implemented in Brainstorm. Source activity was normalized to the baseline. The absolute values of the Z scores were then averaged across participants to obtain grand averaged source maps for each group and condition.

To identify the cortical generators engaged during anticipatory activity, we used a t test and contrasted the individual source maps relative to the averaged widows of interest (i.e., the intervals of significant ERP modulations) separately for each group. The cortical generators showing significant modulations (p < 0.05 uncorrected) are reported on an age-appropriate template cortex smoothed at 40%. A threshold of at least 20 clustered vertices was used to eliminate spurious activations. Importantly, the t test at the source level is only used to properly describe the source distribution of the statistically significant effect established at the sensor level, not for a second statistical test at the source level; therefore, no correction for multiple comparisons is required^16,64^. To anatomically localize the functional activations in the source space, the Desikan-Killany atlas^65^ adapted for EEG cortical source analysis was used. To more accurately depict the time course of the activation of the main cortical ROIs identified, we used the scout analysis tool in Brainstorm. This procedure allows one to cluster subsets of neighboring vertices and to plot their activation values for the temporal dimension.

## Data Availability

The datasets used and/or analysed during the current study available from the corresponding author on reasonable request.
